# Substantivity of mouth-rinse formulations containing cetylpyridinium chloride and O-cymen-5-ol: a randomized-crossover trial

**DOI:** 10.1186/s12903-022-02688-z

**Published:** 2022-12-27

**Authors:** Felipe-Rodrigo Aguilera, Miguel Viñas, Josep M. Sierra, Teresa Vinuesa, Antonio R. Fernandez de Henestrosa, Marta Furmanczyk, Carles Trullàs, Eric Jourdan, José López-López, Marta Jorba

**Affiliations:** 1 Laboratory of Molecular Microbiology and Antimicrobials, Department of Pathology and Experimental Therapeutics, Faculty of Medicine, University of Barcelona & IDIBELL, 08907 L’Hospitalet de Llobregat, Barcelona, Spain; 2 Dental School, Faculty of Medicine, Universidad Austral de Chile, Valdivia, Chile; 3 Department of Dentistry, Faculty of Medicine, University of Barcelona & IDIBELL, Barcelona, Spain; 4 Innovation and Development, ISDIN, Barcelona, Spain

**Keywords:** Mouth-rinses, Substantivity, Salivary microbiota, O-cymen-5-ol, Cetylpyridinium chloride, Prevention, Control

## Abstract

**Background:**

The efficacy of mouth-rinses strongly depends upon their substantivity. The use of natural and non-toxic products that avoid secondary effects is gaining interest in preventive dentistry. The purpose of this study was to evaluate the substantivity of two formulations of mouth-washing solutions based on cetylpyridinium (CPC) and O-cymen-5-ol.

**Methods:**

This was a randomized, double-blind, crossover trial conducted at the Faculty of Medicine and Health Sciences of the University of Barcelona. Bacterial re-colonization was followed by live/dead (SYTO^TM^9 + propidium iodide) bacterial staining and measured by confocal laser scanning microscopy and fluorometry. Unstimulated saliva samples were collected from 16 healthy individuals at baseline saliva and then, at 15 min, 30 min and 1, 2, 3, and 4 h after the following mouth-rinses: (i) a single, 1-min mouth-rinse with 15 ml of placebo (negative control); (ii) a single, 1-min mouth-rinse with 15 ml of CPC (0.05%) ; (iii) a single, 1-min mouth-rinse with 15 ml of O-cymen-5-ol (0.09%); (iv) a single, 1-min mouth-rinse with 15 ml of CPC (0.05%) + O-cymen-5-ol (0.09%).

**Results:**

Proportion of dead bacteria was significantly higher for all mouthrinses during the first 15 min compared to baseline (CPC = 48.0 ± 13.9; 95% CI 40.98–56.99; *p* < 0.001, O-cymen-5-ol = 79.8 ± 21.0; 95% CI 67.71–91.90; *p* < 0.05, CPC + O-cymen-5-ol = 49.4 ± 14; 95% CI 40.98–56.99; *p* < 0.001 by fluorometry and 54.8 ± 23.0; 95% CI 41.50–68.06; *p* < 0.001, 76.3 ± 17.1; 95% CI 66.36–86.14; *p* < 0.001, 47.4 ± 11.9; 95% CI 40.49–54.30; *p* < 0.001 by confocal laser scanning microscopy, respectively). Nevertheless, after 4 h, CPC + O-cymen-5-ol was the only one that obtained significant values as measured by the two quantification methods used (80.3 ± 22.8; 95% CI 67.15–93.50; *p* < 0.05 and 81.4 ± 13.8; 95% CI 73.45–89.43; *p* < 0.05). The combined use of CPC + O-cymen-5-ol increased the substantivity of the mouthrinse with respect to mouthrinses prepared with either of the two active products alone.

**Conclusion:**

The synergistic interaction of CPC and O-cymen-5-ol prolongs their substantivity. The resulting formulation may be as effective as other antimicrobials, such as triclosan or chlorhexidine, but without their undesirable secondary effects. Thus, mouthrinsing products based on Combinations of CPC and O-cymen-5-ol may replace in the near future Triclosan and Chlorhexidine—based mouthrinses.

## Introduction

Oral biofilms are multi-species communities of microorganisms existing as complex ecosystems on both hard and soft tissues of the oral cavity [1–3]. Both the effective removal of biofilms and the prevention of their formation are essential to maintain good oral health [4]. One of the main strategies is to control the formation of young biofilms, as they are much easier to eradicate than mature ones.

Mechanical approaches, such as brushing and flossing, are essential to control dental biofilms [5]. Nevertheless, patients’ efforts to maintain good biofilm control are often hampered by the difficulty of accessing interdental areas and gingival margins [6]. Antimicrobial mouth-rinses complement mechanical oral hygiene regimens by improving biofilm removal [7–9].

Cetylpyridinium chloride (CPC) is a cationic quaternary ammonium compound that has been proposed as an alternative to triclosan and chlorhexidine (CHX), given the latter’s adverse effects, such as tooth staining, taste disturbance, mouth ulcers, burning and even presumptive carcinogenicity [10, 11]. CPC is effective in controlling dental plaque and in preventing gingivitis, based on its significant bactericidal effect on the microorganisms involved in periodontal diseases. While CPC is less bactericidal than CHX, its fewer side effects, mostly related to aesthetics, are the main reason for the increasing interest in its use. The cationic nature of CPC accounts for its antibacterial effect, as it allows binding to negatively charged bacterial surfaces and to the negatively charged proteins of oral tissues [12, 13].

Crude essential oils derived from plants (thyme, oregano, cinnamon, and others) have been classified as GRAS (generally recognized as safe) by the FDA (United States Food and Drug Administration) and accepted as usable in food by the European Union. The use of phytocompounds, including essential oils, as oral disinfectants (or antimicrobials) is gaining interest due to their demonstrated antimicrobial activity and the value currently placed by consumers on natural products [14]. For example, essential oils have been examined for their antibacterial activity in the treatment of periodontitis, including that caused by *Aggregatibacter actinomycetemcomitans* [15]. O-cymen-5-ol (CH_3_)_2_CHC_6_H_3_(CH_3_)OH) is a natural phenolic compound derived from isopropyl cresol and its properties suggest its potential as an agent to maintain buccal health [16–18].

An important quality of mouth rinses is substantivity, defined as the persistence of antimicrobial action in the mouth over time. Substantivity requires adsorption of the agent on buccal surfaces, which implies the late control of biofilm regrowth mostly via bacteriostatic activity [19]. Among the multiple factors that determine substantivity is the control or alteration of adhesion, the duration of the preparation’s antimicrobial activity, and the contributions of synergisms, antagonisms, etc. To improve substantivity and therefore the antimicrobial effectiveness, the addition of different essential oils against microorganisms involved in oral diseases has recently been proposed [20]. Thus, in this work we quantitatively compared the substantivity of oral rinses consisting of: (i) O-cymen-5-ol + CPC, (ii) CPC, and (iii) O-cymen-5-ol in the elimination of salivary microbiota up to 4 h after their use. To our knowledge no previous studies on substantivity of CPC and O-cymen-5-ol have been published.

## Materials and methods

This was a randomized, double-blind, crossover study comparing the in situ persistent antibacterial effect (substantivity) of O-cymen-5-ol + CPC, O-cymen-5-ol, and CPC on the salivary microbiota.

### Selection of the study group

**Fig. 1:**
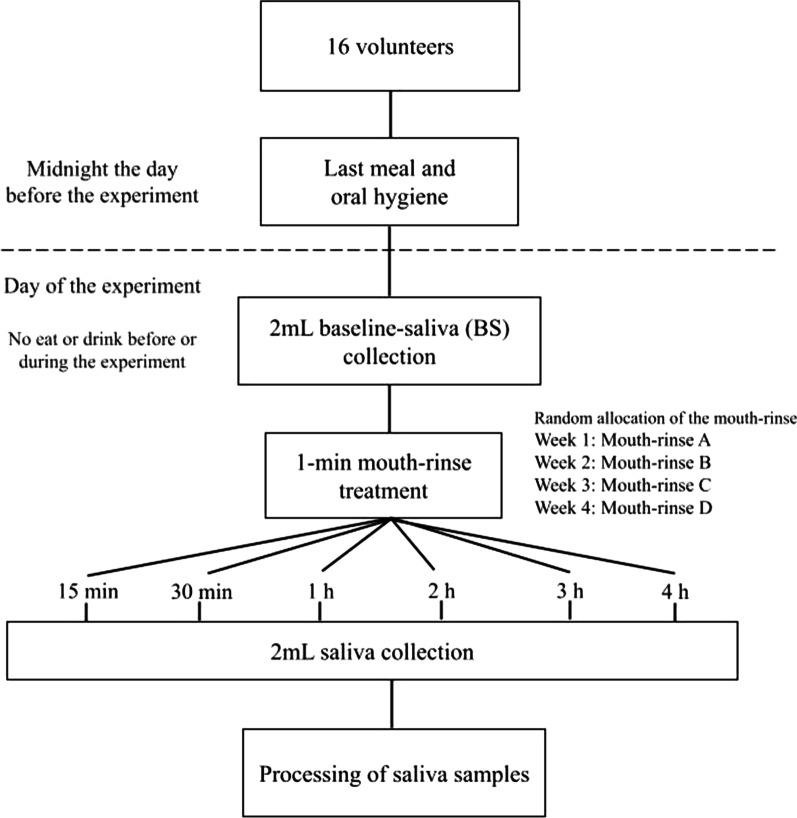
Scheme of the protocol followed by all volunteers of study to later process the saliva samples

The study group comprised 16 adult volunteers between 20 and 45 years of age. All participants had a good oral health status, including a minimum of 24 evaluable permanent teeth with an intact periodontium [pockets ≤ 3 mm and the absence of gingival hemorrhage, according to the criteria of Dietrich et al. [21] and the absence of caries. The exclusion criteria were: smoking, any type of dental prosthesis or orthodontic device, Sjögren syndrome, allergies to oral hygiene products, antibiotic treatment or the routine use of oral antiseptics during the previous 3 months, and the presence of any systemic disease that could lead to an alteration in the production and/or composition of the saliva. All volunteers followed the protocol shown in Fig. 1. Assignment to groups was performed randomly using an Internet-based balanced randomization system (Dallal GE. Available from: www.randomization.com). This study was approved by the Ethics Committee of the Hospital Odontològic Universitat de Barcelona (CEIm, no. 22/2021) and registered at ClinicalTrials.gov PRS, NCT05365737 (09/05/2022).

### Mouthrinse treatment

The volunteers used each of the four mouth-rinses as described below with a one-week resting period between rinses. Written informed consent was obtained from all of the volunteers. Each of the protocols was carried out between May and June 2022 at the Pavellò de Govern (Faculty of Medicine and Health Sciences) of the University of Barcelona.

The volunteers refrained from any type of oral hygiene beginning at midnight the day before the experiment. On the day of the experiment, they were not allowed to eat or drink before or during the experiment. One of the researchers supervised the participants in a living room during the experimental period.

The spitting method [22] was used to collect unstimulated saliva (2 ml) from each volunteer at baseline (BS) and then 15 min, 30 min and 1, 2, 3, and 4 h after the supervised use of the following mouth-rinses: (i) a single, 1-min mouth-rinse with 15 ml of placebo (negative control); (ii) a single, 1-min mouth-rinse with 15 ml of CPC (0.05%); (iii) a single, 1-min mouth-rinse with 15 ml of O-cymen-5-ol (0.09%); and (iv) a single, 1-min mouth-rinse with 15 ml of CPC (0.05%) + O-cymen-5-ol (0.09%).

### Processing of saliva samples

To remove the epithelial cells from the saliva samples and fluidify the mucus, 1 ml of dithiothreitol (Sputolysin^®^, Merck, Darmstadt, Germany) was mixed with the saliva samples (1 ml) in a test tube. After a 1-h incubation at 37 °C, the treated samples were filtered through sterile filters (50-µm pore size; CellTrics^®^, Sysmex, Goerlitz, Germany). The resulting filtrates were then further analyzed.

To establish bacterial viability, the LIVE/DEAD^TM^ BacLight kit (Molecular Probes, Leiden, The Netherlands), which is composed of a cell-permeant cyanine dye (SYTO^TM^9) and propidium iodide (PI), was used. SYTO^TM^9 enters viable and non-viable cells due to its ability to penetrate intact membranes, while PI penetrates only those cells with a high membrane permeability (i.e. damaged cells). Both dyes stain nucleic acids and may be monitored on the basis of their fluorescence. SYTO^TM^9 and PI were mixed at a ratio of 1:1 (6 µL of SYTO^TM^9 and 6 µL of PI in 2 ml of filter-sterilized dH_2_O) and the viability of the treated cells was measured based on fluorometric analysis and confocal laser scanning microscopy (CLSM) as described below.

### Fluorometry

One-hundred µL of the processed sample was pipetted into separate wells of 96-well black microtiter plates; 100 µL of the LIVE/DEAD^TM^ solution was added to the wells using a new tip for each one. Fluorescence was measured using a Fluostar Optima plate reader (BMG Labtech Ortenberg, Germany) based on emission 1 (green), determined at an excitation wavelength of 485 nm and an emission wavelength of 530 nm, and emission 2 (red), using 485-nm and 620-nm filters, respectively. The percent bacterial viability was estimated by comparison with a growth curve derived from a mixture of *Escherichia coli* and *Staphylococcus aureus*, as outlined in the LIVE / DEAD ^TM^ BacLight manual. Green/red fluorescence ratios were calculated for each proportion of live/dead bacteria. The equation to establish viability was y = x * 0.0045 + 0.3383, where x is living bacteria and y is the green/red fluorescence ratio.

### CLSM

One-ml samples were centrifuged in Eppendorf tubes at 10,000 × g for 10 min. The supernatant was discarded while the pellet was resuspended in 100 µl of LIVE/DEAD^TM^ solution and stored in the dark at room temperature for 15 min. Ten µl of the stained bacterial suspension was placed on a slide and covered with a 22 × 22 mm coverslip. Bacterial cells were visualized using a confocal laser scanning microscope (Leica TCS-SL, Leica Microsystems, Mannheim, Germany) equipped with a 488-nm argon laser and 543-nm and 633-nm He/Ne lasers (Centres Científics i Tecnològics, Universitat de Barcelona, Barcelona, Spain). Six fields/samples were selected by the operator (FA) and examined using a 63× oil immersion objective. The image resolution was 1024 × 1024 pixels. Image processing and analysis, including quantification of live and dead bacteria, were performed using the Open Source software project Fiji (Fiji Is Just ImageJ). Cell nuclei and bacterial aggregates were excluded.

### Statistical analysis

The intraclass correlation coefficient (ICC) test was used to determine intra- and inter-observer correlations for CLSM analysis. The mean and standard deviation (%) of viable bacteria were calculated. The percentages were adjusted according to the baseline, set as 100%. A repeated-measures ANOVA and simple comparisons were used between groups and time points. Differences were considered statistically significant at a p-value < 0.05. The data were statistically analyzed using Rstudio (v1.2.1335 for Mac OS).

## Results

**Fig. 2:**
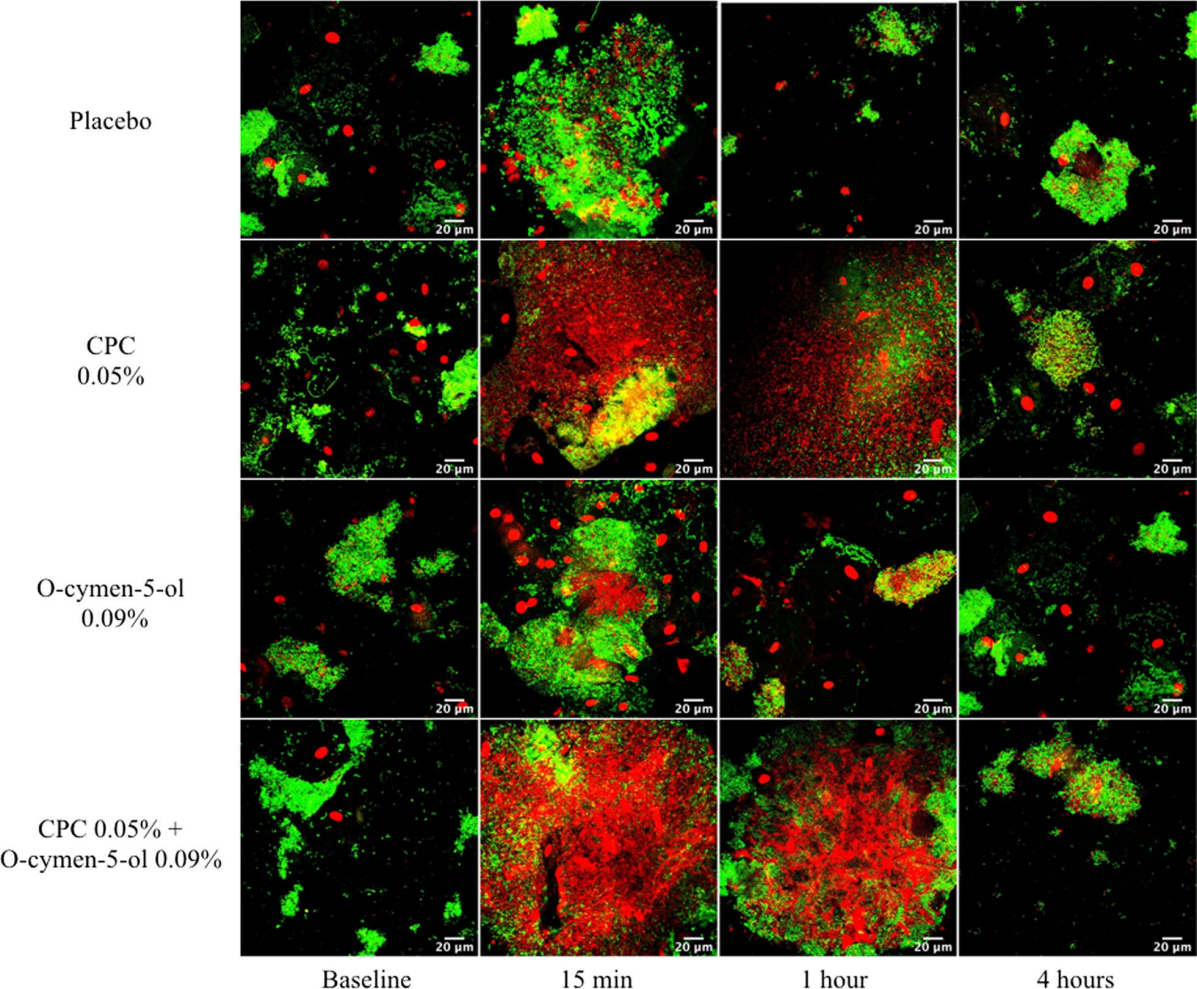
Representative micrographs of bacterial viability at baseline and 15 min, 1 h, and 4 h after a single use of mouth-rinse: placebo, CPC (0.05%), O-cymen-5-ol (0.09%) and CPC (0.05%) + O-cymen-5-ol (0.09%). Note: Green: viable bacteria; red: dead bacteria; large red cells: epithelial cells

Both CLSM visualization and the subsequent quantification of viable and dead bacteria showed differences in bacterial viability over time between mouth-rinses (Fig. 2).

In the intra- and inter-observer correlation analysis of the CLSM results, the mean ICC was 0.85 (*p* < 0.001) and 0.80 (*p* < 0.001) respectively.

**Fig. 3:**
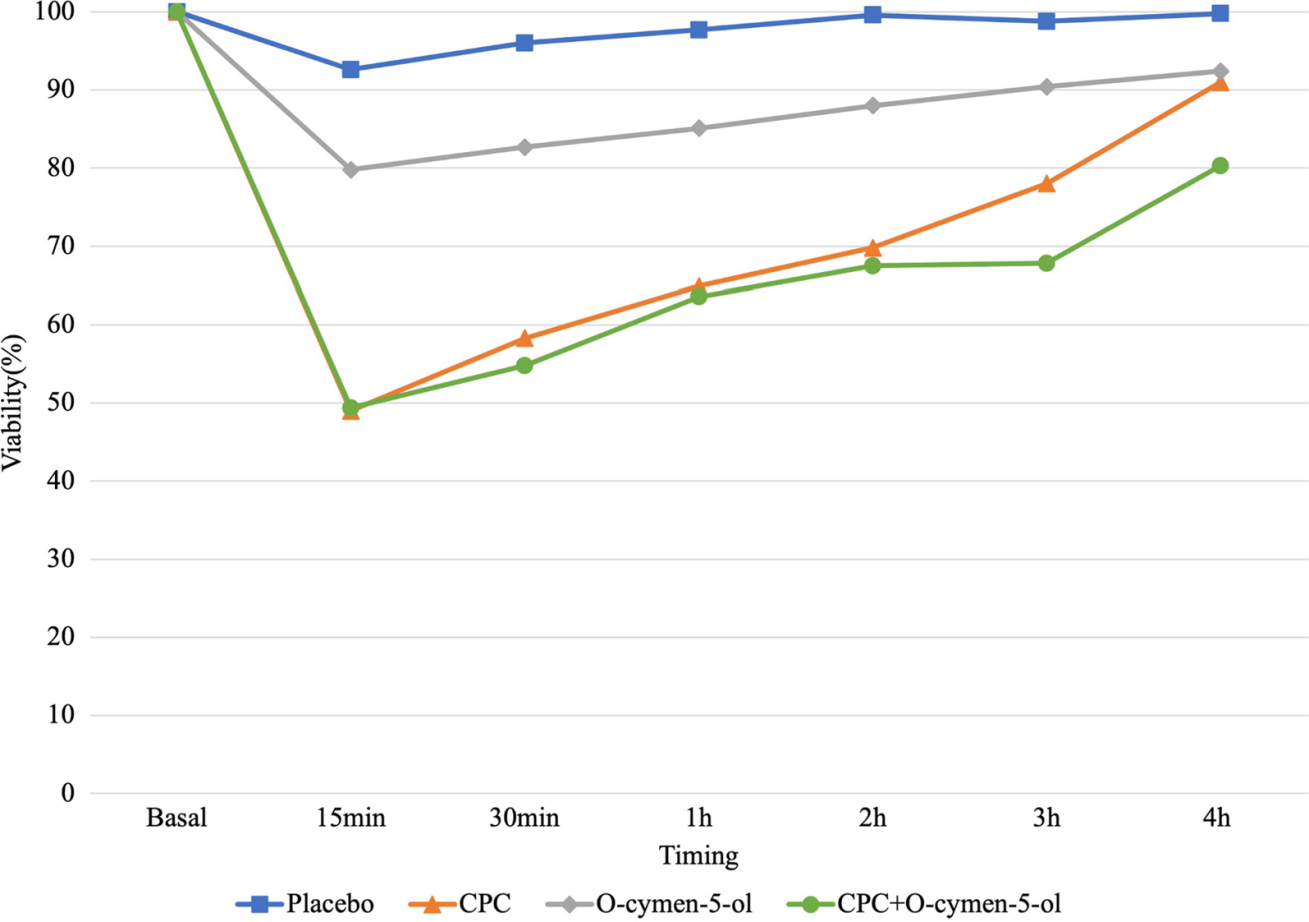
Mean bacterial viability (%) in saliva at baseline and at different times after a single use of mouth-rinse or placebo, determined using fluorescence

**Fig. 4:**
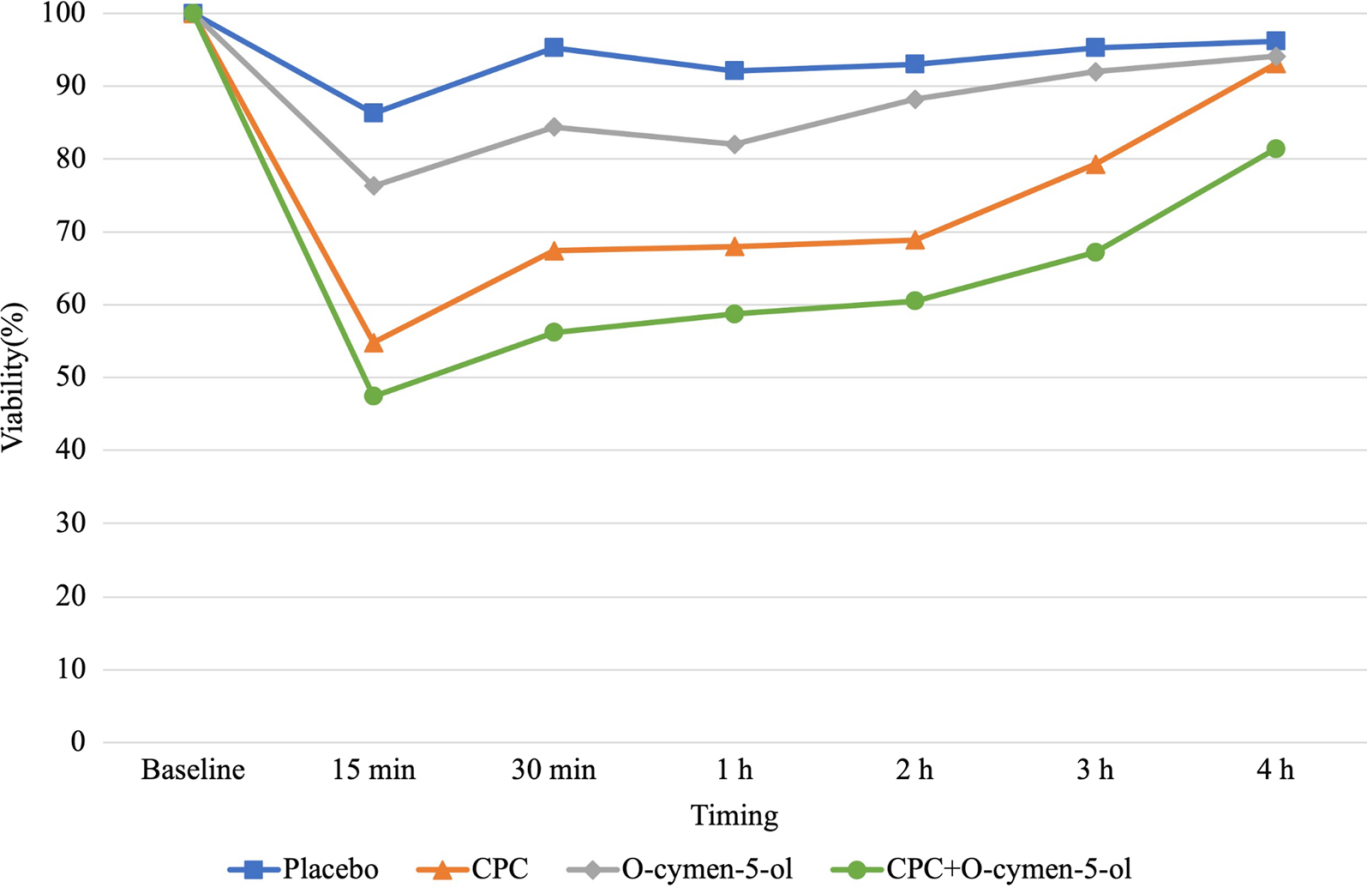
Mean bacterial viability (%) in saliva at baseline and at different times after a single use of mouth-rinse or placebo, determined using confocal laser scanning microscopy

The largest decrease in salivary bacterial viability was obtained after a single application of the mouth-rinse containing CPC + O-cymen-5-ol, followed by that containing CPC alone, O-cymen-5-ol alone, and placebo (Figs. 3 and 4). The results of the analyses of bacterial viability and of the effect of the mouth-rinses on the salivary microbiota, tested at different times using fluorometry and CLSM, are shown in Tables 1 and 2, respectively.

**Table 1:** Intra-mouthrinse comparisons of viability of salivary microbiota (%), measured as fluorescence, after treatment with placebo (sterile water), CPC, O-cymen-5-ol, and CPC + O-cymen-5-ol

Time	Mouthrinse	Mean ± SD	95% Conf. Interval	*p*-value
15 min	Placebo	92.6 ± 18.7	80.03–105.12	0.38
	CPC	48.0 ± 13.9	40.98–56.99	< 0.001
	O-Cymen-5-ol	79.8 ± 21.0	67.71–91.90	< 0.05
	CPC + O-Cymen-5-ol	49.4 ± 14.1	40.98–56.99	< 0.001
30 min	Placebo	96.0 ± 15.6	85.52–106.52	0.64
	CPC	58.3 ± 12.9	50.80–65.71	< 0.001
	O-Cymen-5-ol	82.7 ± 23.0	69.46–95.98	< 0.05
	CPC + O-Cymen-5-ol	54.8 ± 22.9	41.60–68.02	< 0.001
1 h	Placebo	97.7 ± 23.6	81.85–113.59	0.80
	CPC	64.9 ± 13.6	57.04–72.75	< 0.001
	O-Cymen-5-ol	85.1 ± 21.9	72.45–97.76	0.05
	CPC + O-Cymen-5-ol	63.6 ± 21.1	51.42–75.82	< 0.001
2 h	Placebo	99.6 ± 19.3	86.65–112.62	0.98
	CPC	69.8 ± 17.0	60.03–79.67	< 0.001
	O-Cymen-5-ol	88.0 ± 28.7	71.44–104.55	0.12
	CPC + O-Cymen-5-ol	67.5 ± 16.4	58.03–76.94	< 0.001
3 h	Placebo	98.8 ± 19.1	86.00–111.69	0.91
	CPC	78.0 ± 14.0	69.92–86.09	< 0.05
	O-Cymen-5-ol	90.4 ± 27.5	74.53–106.23	0.22
	CPC + O-Cymen-5-ol	67.8 ± 25.7	52.94–82.65	< 0.001
4 h	Placebo	99.8 ± 17.5	87.97–111.52	0.93
	CPC	91.0 ± 25.5	76.28–105.74	0.25
	O-Cymen-5-ol	92.4 ± 26.7	76.91–107.81	0.34
	CPC + O-Cymen-5-ol	80.3 ± 22.8	67.15–93.50	< 0.05

*ANOVA with repeated measures was used to calculate the *p*-value

*SD * Standard deviation

**Table 2:** Intra-mouthrinse comparisons of viability of salivary microbiota (%), measured under CLSM, after treatment with placebo (sterile water), CPC, O-cymen-5-ol, and CPC + O-cymen-5-ol.

Time (in min)	Mouthrinse	Mean ± SD	95% Conf. Interval	*p*-value
15 min	Placebo	86.3 ± 10.7	79.14–93.48	0.09
	CPC	54.8 ± 23.0	41.50–68.06	< 0.001
	O-Cymen-5-ol	76.3 ± 17.1	66.36–86.14	< 0.001
	CPC + O-Cymen-5-ol	47.4 ± 11.9	40.49–54.30	< 0.001
30 min	Placebo	95.3 ± 16.0	84.53–106.01	0.86
	CPC	67.4 ± 22.7	54.25–80.44	< 0.001
	O-Cymen-5-ol	84.4 ± 12.9	76.97–91.89	< 0.05
	CPC + O-Cymen-5-ol	56.2 ± 12.7	48.88–63.49	< 0.001
1 h	Placebo	92.1 ± 5.9	88.15–96.09	0.48
	CPC	68.0 ± 17.0	58.20–77.83	< 0.001
	O-Cymen-5-ol	82.0 ± 12.4	74.80–89.10	< 0.05
	CPC + O-Cymen-5-ol	58.7 ± 8.9	53.54–63.87	< 0.001
2 h	Placebo	93.0 ± 11.1	85.54–100.52	0.58
	CPC	68.9 ± 19.5	57.65–80.14	< 0.001
	O-Cymen-5-ol	88.2 ± 8.0	83.57–92.80	0.14
	CPC + O-Cymen-5-ol	60.5 ± 18.0	50.11–70.86	< 0.001
3 h	Placebo	95.3 ± 8.0	89.94–100.69	0.87
	CPC	79.3 ± 18.5	68.63–90.06	< 0.001
	O-Cymen-5-ol	92.0 ± 9.9	86.34–97.73	0.44
	CPC + O-Cymen-5-ol	67.2 ± 11.8	60.44–74.05	< 0.001
4 h	Placebo	96.2 ± 8.3	90.63–101.84	0.88
	CPC	93.1 ± 22.8	79.95–106.29	0.57
	O-Cymen-5-ol	94.1 ± 9.1	88.84–99.40	0.70
	CPC + O-Cymen-5-ol	81.4 ± 13.8	73.45–89.43	< 0.05

*ANOVA with repeated measures was used to calculate the *p*-value

*SD* Standard deviation

Fluorometry revealed significant differences when compared the three mouth rinses to BS; following the use of the CPC and CPC + O-cymen-5-ol during 3 and 4 h, respectively (*p* < 0.05). O-cymen-5-ol alone had significant effect only during the initial 30 min. Similar results were obtained with CLSM. Nevertheless, O-cymen-5-ol decreased alive bacteria proportion significantly during the first hour (*p* < 0.05).

Comparisons between mouth-rinses also showed significant differences for CPC and CPC + O-cymen-5-ol compared to placebo during the first 2 and 3 h, respectively (*p* < 0.05). On the contrary, O-cymen-5-ol did not significantly differ from placebo (*p* > 0.05) irrespective of the time point and measurement method.

## Discussion

Essential oils have interesting antimicrobial and other bioactivities including, antiviral, antioxidant, and anticancer activities [23]. Their main use thus far has been in food technologies, with recent expansions into pharmaceuticals and cosmeceuticals, reflecting the rapidly growing demand by consumers for “natural products.” There has also been a move to replace CHX and triclosan with CPC, especially after CPC was shown to inactivate the COVID-19 coronavirus in the oral cavity [24]. Accordingly, both O-cymen-5-ol and CPC have garnered interest as replacements for traditional mouth-rinses with antibacterial activity.

Substantivity is a critical property of mouth-rinses. Typically, the efficacy of antiseptics is evaluated in in vitro suspensions or in other specifically designed tests or in tests similar to those used to assess antibiotics. However, the substantivity (persistence) of an antimicrobial cannot be quantified in vitro but must necessarily be investigated in vivo. In this study, we evaluated and compared the substantivity of two putative components of the new generation of mouth-rinses, alone and in combination.

The substantivity of CPC-containing mouth-rinses on the salivary microbiota has been examined in a few reports. Elworthy et al. [19] found that the tested CPC rinses were similar and all were significantly more substantial than the control rinses between 180 and 300 min. The same authors found that additional components, such as fluoride and/or alcohol, did not substantially enhance the activity of CPC. In spite we failed in demonstrating synergistic effect of CPC and O-cymen 5 ol in terms of Minimal inhibitory concentrations (data not shown), we succeed in demonstrating that the combination of active molecules did maintain bacterial counts at a lower level and for a longer period of time than BS.

Similar results were obtained by Roberts & Addy [25], who compared the antibacterial properties of three antiseptic mouth-rinses, containing CHX, CPC, and hexetidine, and of an alexidine preparation. A return to pre-rinse levels after 90 min was obtained with hexetidine, after 3 h with CPC, after 5 h with alexidine, and after 7 h with CHX gluconate. Residual salivary antibacterial activity remained for 90 min with CPC, for 3 h with hexetidine and alexidine, and for 5 h for CHX gluconate.

Epifluorescence microscopy has been used to assess the effect of CHX on the salivary microbiota. Quintas et al. [26] evaluated the immediate effect of a 0.2% CHX mouthwash and found a significant reduction in bacterial viability compared to BS for up to 7 h (87.6 ± 6.5% vs. 73.6 ± 8.8%; *p* < 0.001). Tomás et al. [27] reported significantly lower counts of viable salivary bacteria 30 s after a 0.2% CHX rinse than after the control sterile water treatment (*p* < 0.001), with a significant antibacterial effect achieved for up to 7 h with the former (*p* < 0.001). Our investigation showed significant (*p* < 0.05) reductions in salivary bacteria for up to 4 h following mouth-rinse treatments with CPC and O-cymen-5-ol, as demonstrated by fluorometry and CLSM.

CHX has long been the gold standard in dental mouth-rinses, based on its bactericidal properties against gram-negative and gram-positive species, yeast, and other microorganisms. However, CHX use is associated with a high incidence of adverse effects, such as tooth enamel staining, taste disturbance, and the appearance of ulcers in the mouth and tongue [12]. In addition, there is evidence of the potential resistance of oral bacteria to CHX when used as a standard antiseptic [28]. Consequently, alternative mouth-rinse formulations, such as those based on CPC and complemented by the addition of natural products, such as O-cymen-5-ol, with demonstrated substantivity should be explored.

In this research, the LIVE ⁄ DEAD ^TM^ assay was used to quantify bacterial viability. Its advantages include speed, simplicity, and the simultaneous quantification of viable and non-viable bacteria. However, the assay may determine higher percentages of nonviable bacteria than in the actual sample [29]. However, Tawakoli et al. [30] demonstrated the validity of the LIVE/DEAD^TM^ assay in estimating bacterial viability and that the combination of SYTO^TM^9 / PI is a reliable tool in the evaluation of multi-species biofilms. Our results showed that the LIVE/DEAD^TM^ assay can be used to analyze the effects of antimicrobials that alter the integrity of the cytoplasmic membrane in different oral ecosystems.

Among the limitations of our study was the quantification of bacterial viability by CLSM, as errors in the counts may have arisen due to bacterial aggregation, the staining of cellular elements, or the presence of contaminating material. Furthermore, microscopic examination is tedious since a large number of fields and samples must be assessed. Nevertheless, the results have been confirmed by fluorometric automatic reading emphasizing the validity of the general conclusion. In addition, in future research the substantivity of other CPC additives, tested at different concentrations, should be measured.

The main limitations of this work reside in the nature of the samples and in the possible heterogeneity of the oral bacterial populations of the different participants. The saliva carries bacteria that originate from planktonic and sessile states, so the data must be interpreted as a combined effect of the washers on both populations. In any case, the collection of other types of samples, such as biofilm scraping, also entail numerous biases due to the collection methods. On the other hand, the only data handled are those of bacteria with intact membranes (and therefore presumably live bacteria) and those that have their membranes damaged and are therefore dead bacteria. In any case, the approximation seems adequate and has already been used on other studies. The maximum times of four hours were taken based on the data obtained in pilot tests carried out prior to the beginning of the experiment. Also we decided to leave a week of rest between tests to be able to use the same volunteers and after verifying that after a week, the oral microbiota values ​​were apparently fully restored.

In summary, our study showed a higher substantivity of the mouth-rinse CPC + O-cymen-5-ol or CPC alone than of O-cymen-5-ol alone (or placebo). The addition of O-cymen-5-ol extended the substantivity of CPC by more than 1 h (from 3 to 4 h) and significantly reduced bacterial recovery compared to baseline. Compared to placebo, substantivity was increased by the addition of O-cymen-5-ol to CPC, from 2 h (CPC) to 3 h (CPC + O-cymen-5-ol). These observations demonstrate the synergy of CPC and O-cymen-5-ol in increasing substantivity.

## Data Availability

Data will be made available on a case-by-case basis and also additional information will be provided by contacting the Corresponding Author.
